# Can physical exercise modify intestinal integrity and gut microbiota composition? A systematic review of in vivo studies

**DOI:** 10.5114/biolsport.2025.148545

**Published:** 2025-04-14

**Authors:** Stephanie Michelin Santana Pereira, Vinícius Parzanini Brilhante de São José, Alessandra da Silva, Karina Vitoria Cipriana Martins, Luciano Bernardes Leite, Pedro Forte, Antônio José Natali, Hércia Stampini Duarte Martino, Ceres Mattos Della Lucia, Josefina Bressan

**Affiliations:** 1 Laboratory of Vitamin Analysis, Department of Nutrition and Health, Universidade Federal de Viçosa, MG, Brazil; 2 Laboratory of Experimental Nutrition, Department of Nutrition and Health, Universidade Federal de Viçosa, MG, Brazil; 3 Laboratory of Energy Metabolism and Body Composition, Department of Nutrition and Health, Universidade Federal de Viçosa, MG, Brazil; 4 Exercise Biology Laboratory, Department of Physical Education, Universidade Federal de Viçosa, MG, Brazil; 5 Department of Sports, Instituto Politécnico de Bragança, Bragança, Portugal; 6 Department of Sports, Higher Institute of Educational Sciences of the Douro, Penafiel, Portugal; 7 CI-ISCE, ISCE Douro, Penafiel, Portugal; 8 Research Center for Active Living and Wellbeing (LiveWell), Instituto Politécnico de Bragança, Bragança, Portugal

**Keywords:** Microbiome, Microbial community compositions, Physical activity, Training, Review

## Abstract

There is little evidence about how physical exercise affects the gut microbiota since studies in the field are relatively recent. Thus, we aimed to systematically review the main effects of regular physical exercise on the intestinal integrity and microbiota composition in animal models, discuss the mechanisms involved, and indicate future directions. Searches for original articles were performed in PubMed/MEDLINE, Scopus, Web of Science, and Embase. A total of 18 studies were selected. These studies suggest that physical exercise has a significant impact on the gut microbiota. Voluntary running increased the thickness of duodenal villi and microbiota diversity but reduced its richness. Low-intensity treadmill running increased the abundance of the phylum Actinobacteria and the family Bifidobacteriaceae, while that of moderate-intensity reduced the Bacteroides/Prevotella ratio. High-intensity swimming and treadmill running altered the gut microbiota, evidenced by β-diversity, and increased the Shannon and Chao indices but reduced short-chain fatty acids. Resistance exercise increased the Chao index and altered the functionality of the gut microbiota, increasing carbohydrate metabolism and reducing lipid and amino acid metabolism. Thus, regular physical exercise of different intensities and types can modify the gut microbiota, and the exercise benefits appear to be positively associated with training intensity.

## INTRODUCTION

Microbiota is the set of microorganisms that inhabit an environment [1]. A population of more than 100 trillion bacteria is found in humans and experimental animals, which act in the modulation of metabolic pathways in their host [2, 3].

Several studies have demonstrated the essential role of the microbiota in modulating metabolic pathways, including digestion, nutrient metabolism, regulation of the immune system, and development of intestinal epithelial tissue, in addition to acting in the prevention of chronic diseases and production of bioactive compounds, such as short-chain fatty acids (SCFA) [4–6]. However, the composition of the microbiota can change, disrupting the correct functioning of these mechanisms [7, 8]. The change in the composition and diversity of the microbiota may occur due to chemical components, immunological activity, and lifestyle factors like diet and physical activity, among others [9].

In mice, the practice of highly intensive physical exercises can increase the diversity and abundance of beneficial bacteria, such as Bacteroidetes, which plays a crucial role in several metabolic pathways like the degradation of complex sugar polymers, the improvement of glucose metabolism, and branch-chain amino acid degradation [5, 10]. In addition, physical exercise has been shown to increase mitochondrial function, resulting in the development of essential bacteria for urease production and lactate metabolism, processes relevant to the performance and recovery of athletes [11]. However, physical exercise was reported to interfere with the balance of the microbiota, leading to increased intestinal permeability and the Firmicutes/Bacteroidetes ratio, as well as a possible increase in food intake and weight gain [12].

Understanding the mechanisms underlying the influences of regular physical exercise in the microbiota and vice versa is of interest to athletes since the modulation of the gut microbiota can improve the use of nutrients and sports performance and reduce recovery time during training and competitions [13]. For practitioners of general physical activities, the modulation of the gut microbiota can reduce the risk of chronic diseases [14]. Thus, this study aimed to systematically review and analyze the main effects of regular physical exercise on intestinal integrity and gut microbiota composition in animal models. Furthermore, we sought to explore the underlying mechanisms involved in these interactions, address the challenges and limitations in current research methodologies, and identify key directions for future studies in the fields of nutrition science and physical exercise. This comprehensive approach aims to contribute to a deeper understanding of the complex relationship between physical activity and gut health.

## MATERIALS AND METHODS

### Protocol and registration

This systematic review was based on the following question: “Can physical exercise modify intestinal integrity and the composition of the gut microbiota?” The Preferred Reporting Items for Systematic Reviews and Meta-Analyses (PRISMA) guidelines were used to report the systematic review [15]. The review was registered in the International Prospective Register of Systematic Reviews (PROSPERO) (Register number: CRD42023420063).

### Eligibility criteria

In this review, we included *in vivo* studies with healthy rats or mice. Eligibility criteria were original studies that evaluated the effect of voluntary or forced physical exercise, with any follow-up time, on gut microbiota markers such as composition, diversity, and functionality. The results were compared to the control group, i.e., the group without physical exercise.

The use of animal models in this review was based on several scientific and methodological reasons. First, animal studies allow for rigorous control of experimental variables, such as diet, physical activity level, and environmental conditions, providing a more accurate view of the isolated effects of physical exercise on the gut microbiota. Furthermore, despite the differences between the physiology and microbiota composition of humans and animals, findings in animal models often provide important initial insights that can later be validated in clinical studies.

Finally, the choice to prioritize animal models reflects the significant gap in the literature on the detailed interaction mechanisms between physical exercise and gut microbiota. Human studies, although promising, still face challenges such as genetic variability, protocol adherence, and self-reporting bias, limiting their applicability to investigate more complex mechanisms.

Review articles, book chapters, studies that did not assess the markers of interest, and studies carried out with animals other than rats or mice were not included. The population, intervention or exposure, comparator, outcomes, and study design (PICOS) used for this study are shown in Table 1.

**TABLE 1:** PICOS criteria for the inclusion of studies

Parameter	Inclusion criteria
Population	*In vivo s* tudies using rats and mice.
Intervention or exposure	Physical exercise (forced or voluntary and regardless of duration).
Comparison	Control (without physical exercise).
Outcome	Intestinal barrier integrity and gut microbial community analysis.
Study design	Experimental studies.

### Search

Searches were conducted across multiple databases, including MEDLINE (via PubMed PubMed/MEDLINE https://www.ncbi.nlm.nih.gov/pubmed), Scopus (https://www.scopus.com/home.uri), Embase (https://www.embase.com), and Web of Science (https://www.webofknowledge.com). Publication date and language were not used as restriction criteria.

A combination of the following search terms was performed: microbiome, gastrointestinal microbiome, microbial community, microbial community composition, microbial community structure, gut microbiome AND exercises, physical activity, physical exercise, acute exercise, isometric exercises, aerobic exercise, exercise training (Table SI). The search in PubMed was prepared according to the hierarchical distribution of MeSH terms (Medical Subject Headings), which was adapted for other platforms. The last search was performed on April 2, 2024.

The references and citations of selected articles (gray literature) were used to find other relevant studies. Also, the snowball method was used to find national articles. After that, the authors read and compared the articles to resolve the differences.

### Selection of studies

Four authors (SMSP, VPBSJ, KVCP, and LBL) independently selected the after reading the titles and abstracts, with a final level of agreement assessed by the kappa index of 0.93. Afterward, they were read in full, and the *in vivo* studies that evaluated the effect of performing or not performing physical exercise on the composition and integrity of the microbiota were included. For the removal of duplicates and blind reading by the authors, the Rayyan software was used [16]. Differences among the authors were resolved in consultation with another reviewer (AS) and the entire work was guided and reviewed by the other authors (AS, PF, AJN, HSDM, CMDL, JB).

### Data extraction

After selecting the articles, the following data were extracted in a standardized and independent way: reference, year of publication, animal model, study protocol, duration, intervention characteristics, and the results of interest. Then, both authors analyzed these data to guarantee the study’s reliability and integrity (Table 2).

**TABLE 2:** Characteristics of the studies included.

Author, year	Animal model	Study protocol	Follow-up	Intervention characteristics	Main results
**Queipo-Ortuño et al., 2013 [24]**	5-wk-old male Sprague Dawley rats	G1: Exercise group (n = 10) G2: Sedentary group (n = 10)	6 days	Voluntary running (Free access to running wheel)	↑ Genus: *Bacteroides, Prevotella, Clostridium,* and *Helicobacter* in G2 *vs*. G1; Genus: *B. Coccoides-E rectale, Lactobacillus* and *Bifidobacterium,* Phylum: Actinobacteria and Bacteroidetes in G1 *vs*. G2. ↓ Phylum: Firmicutes; Genus: *Enterococcus* in G1 *vs*. G2.

**Allen et al., 2015 [18]**	6-wk-old male C57BL/6J mice	FTR group (n = 10) VWR group (n = 10) SED group (n = 9)	6 weeks	Treadmill running (Intensity: 8–12 m/min, 5% grade); 40 min/session; 1 session/ day; 5 days/wk. Voluntary running (Free access to running wheel)	↔ Species richness in the feces between the groups by Shannon index; Bacteroidetes and Firmicutes. ↑ Species richness in cecal contents in VWR by Shannon index; Tenericutes and Proteobacteria in FTR (FTR *vs*. SED/ VWR); Genus: *Dorea* in FTR (FTR *vs*. SED), *Anaerotruncus* in VWR (VWR *vs*. SED). ↓ Species richness in both the feces and the cecal contents in VWR by Chao1; Genus: *Turicibacter* in VWR (VWR *vs*. SED). * The exercise modified the beta diversity.

**Lambert et al., 2015 [21]**	6-wk-old male C57BL/KsJ mice	G1: Sedentary group (n = 10) G2: Exercise group (n = 10)	6 weeks	Treadmill running (Intensity: 2.87–4.79 m/min, 0% grade); 60–66 min/session; 1 session/day; 5 days/wk.	↔ Total bacteria and *Enterobacteriaceae family*. ↑ Specie: *Bifidobacterium spp., Lactobacillus spp., Clostridium leptum* and *Clostridium cluster* (C-I) in G2. ↓ Genus: *Bacteroides/Prevotella spp.* and *Methanobrevibacter spp*. in G2.

**Mika et al., 2015 [23]**	Male Fischer F344 rats Juvenile: 24 postnatal days Adult: 70 postnatal days	G1: Juvenile sedentary group (n = 10) G2: Adult sedentary group (n = 10) G3: Juvenile run group (n = 10) G4: Adult run group (n = 10)	6 weeks	Voluntary running (Free access to running wheel)	↔ Microbial communities and species between G2 and G4. ↑ Microbial communities balanced (G2 and G4); Species, Phylum Actinobacteria in adult groups (Adults *vs*. Juvenile); Phylum Euryarchaeota and Bacteroidetes, Genus *Blautia spp.,* *Anaerostipes spp.* and *Methanosphaera spp.* in G3; Genus *Rikenellaceae g_AF12, Rikenellaceae g_* and *Turicibacter spp.* in G4. ↓ Microbial communities balanced in G3 (G1 *vs*. G3); Species in run groups (Run *vs*. Sedentary); Species, Phylum Firmicutes and Proteobacteria in G3 (G1 *vs*. G3); Genus: *Rikenellaceae g_AF12, Rikenellaceae g_* and *Desulfovibrio spp*. in G3. * Dissimilarity of microbial communities between the G1 and the G3 group.

**Campbell et al., 2016 [20]**	6-wk-old male C57BL/6NTac mice	LX: Lean exercise group (n = 9) LS: Lean sedentary group (n = 9)	12 weeks	Voluntary running (Free access to running wheel)	↔ Occludin expression, COX-2 expression. ↑ Thickness of villi, Specie: *Allobaculum* spp. and *Faecalibacterium prausnitzii*, Order: *Clostridiales* in LX. ↓ E-cadherin expression in LX.

**Batacan et al., 2017 [19]**	12-wk-old male Wistar rats	LIT: Light intensity trained group (n = 3) HIIT: High-intensity interval trained group (n = 6) CLT: Control group (n = 6) SED: Sedentary group (n = 8)	12 weeks	Treadmill running (Intensity: 8 m/min, 0% grade), 4 × 125 min with 2 h interval/day; 5 days/wk. Treadmill running (Intensity: 50 m/min, 10% grade), 4 × 150 s with 3 min resting interval/session; 1 session/day; 5 days/wk.	↔ Phylum level, alpha diversity between activity groups. ↑ Beta diversity difference between groups; Tenericutes (Family *Erysipelotrichaceae*) and Actinobacteria (Families *Bifidobacteriaceae* and *Coriobacteriaceae*), Specie: *Parasutterella excrementihominis* and *Lactobacillus johnsonii* in LIT; Specie: *Clostridium saccharolyticum* in HIIT; Specie: *Clostridium geopurificans* in HIIT and LIT.

**Lamoureux et al., 2017 [31]**	6- to 10-wk-old male and female C57BL/6 mice	VE: Voluntary exercise group (n = 10) FE: Forced exercise group (n = 11) VC: Sedentary control group (n = 21)	55 days	Voluntary running (Free access to running wheel) Treadmill running, (Intensity: 15–20 m/min, 0% grade); 40 min/session; 5 days/wk.	↔ Alpha diversity; Species richness for mucosal samples; Beta-diversity; Phylum: Proteobacteria and Family: *Bacteroidaceae* (VE *vs*. VC); Phylum: Bacteroidetes (FE *vs*. VC). ↑ Phylum: Bacteroidetes, Firmicutes, Actinobacteria and Family: S24-7 and Order: *Clostridiales* (VE *vs*. VC); Genus: *Bacteroides* (FE *vs*. VC). ↓ Family: *Rikenellaceae* (VE *vs*. VC); Phylum: Firmicutes and Genus: *Parabacteroides* and Order: *Clostridiales, Lactobacillales* (FE *vs*. VC).

**Yuan et al., 2018 [28]**	6-wk-old male Kunmin mice	ES: Chow diet plus excessive swimming group (n = 10) NS: Chow diet plus non-swimming group (n = 10)	4 weeks	Swimming Two sessions/day (morning and afternoon) of swimming until exhaustion; 7 days/wk.	↔ Small intestine and colon structures. ↑ Abundant phyla: Bacteroidetes and Firmicutes; Abundant family: *Helicobacteraceae*; Abundant genera: *Helicobacter, Bacteroides* in ES. ↓ Overall diversity of the microbiota; Abundant phyla: Proteobacteria; Abundant family: *Bacteroidales_S24-7_group, Lachnospiraceae*; Abundant genera: *Odoribacter* in ES. * Dissimilarity of bacterial communities between the NS and the ES group.

**Wang et al., 2020 [10]**	12-month-old ICR mice	G1: Control plus AIN-93M diet group (n = 9) G2: High-intensity interval training plus AIN-93M diet group (n = 9)	7 weeks	Treadmill running (Intensity: 90%$\dot{\text{V}}0_{2\max}$, 0% grade); intermittent run (5 × 3 min to 6 × 4 min with 2-min resting intervals/session); 1 session/day; 5 days/wk.	↑ Shannon index; Phylumml: TM7 and Genus: *Dorea* and *Dehalobacterium*; Functions related to carbohydrate metabolism in G2. ↓ Phylum Proteobacteria and *Candidatus* *arthromitus* genera; Genes related to diseases in G2. * Beta diversity analysis showed differences between the groups.

**Castro et al., 2021 [2]**	45-day-old male Wistar	G1: Sedentary control group G2: Resistance training group	12 weeks	Resistance training (Intensity: progressive till exhaustion); over 4 climbings on a vertical ladder/session; 1 session/ day; 3 days/wk.	↑ Chao index in the G2 (G1 *vs*. G2); Genus: *Coprococcus_1* relative abundance. ↓ Genus: *Pseudomonas, Serratia* and *Comamonas* relative abundance, lipid and amino acids metabolism, cellular processes and signaling in G2. * Beta diversity analysis showed differences between G2 compared to G1 and G2 baseline.

**Meliala et al., 2021 [22]**	3- to 4-month-old male Wistar rats	G1: Control group (n = 6) G2: Moderate-intensity physical exercise group (n = 6) G3: High-intensity physical exercise group (n = 6)	5 weeks	Treadmill running G2: (Intensity: 55% $\dot{\text{V}}0_{2\max}$, 10% grade); 30 min/ session; 1 session/day; 5 days/wk. G3: (Intensity: 85% $\dot{\text{V}}0_{2\max}$, 10 % grade); 30 min/ session; 5 days/wk.	↔ SCFA production in G2 (G1 *vs*. G2); Lactic acid bacteria among the groups. ↓ SCFA production in G3 (G1 *vs*. G3); Specie: *Escherechia coli* in G2 (G2 *vs*. G3).

**Soares et al., 2021 [25]**	140-day-old male Wistar rats	G1: Sedentary plus commercial diet group (n = 10) G2: Trained plus commercial diet group (n = 10)	4 weeks	Treadmill running (Intensity: 15 m/min, 10% grade); 30 min/session; 1 session/day; 5 days/wk.	↔ Bacteria count, colon histology. ↑ Propionic acid on the gut and feces in G2. ↓ Succinic acid in the gut and acetic acid in the feces in G2.

**Yang et al., 2021 [27]**	5-wk-old male C57BL/6 mice	G1: No exercise group (n = 15) G2: Exercise group (n = 15)	14 weeks	Treadmill running (Intensity: 14–19 m/min, 0% grade); 60 min/session; 1 session/day; 5 days/wk:	↔ Beta diversity. ↑ Bacteroidetes, metabolic activity was related to the increase of microbiota in G2; Chao index in G1.

**Anhê et al., 2022 [30]**	2- to 26-month-old male and female C57BL/6J mice	Y-SED: Young sedentary group (n = 39) O-SED: Old sedentary group (n = 26) O-AET: Old lifelong aerobic exercise group (n = 30)	24 months	Voluntary running (Free access to running wheel)	↔ Beta diversity (O-SED *vs*. O-AET) ↓ Family: *Lachnospiraceae, Turicibacteriaceae*, Genus: *Allobaculum, Anaeroplasma* and *Akkermansia* in O-AET (O-SED *vs*. O-AET); Genus: *Eubacterium* in O-AET (O-AET *vs*. Y-SED).

**Chen See et al., 2022 [32]**	16-wk-old C57/WT mice	G1: No exercise group (n = 12) G2: Exercise group (n = 11)	8 weeks	Treadmill running, (Intensity: 15 m/min, 0% grade); 30 min/session; 1 session/day; 5 days/wk.	↔ Alfa diversity; Firmicutes/Bacteroidetes ratio. * Beta diversity analysis showed differences between the groups.

**Tansathitayaet al., 2022 [12]**	8- to 11-wk-old male Sprague-Dawley rats	G1: No exercise group (n = 6) G2: Light exercise group (n = 6) G3: Heavy exercise group (n = 6)	45 days	Treadmill running (60 min/session; 1 session/ day; 5 days/wk). Light (Intensity: 10–15 m/ min, 0% grade). Heavy (Intensity: 20–25 m/ min, 0% grade).	↑ Chao 1 index in G3 (G2 *vs*. G3). * Beta diversity analysis showed differences among exercise groups compared to G1. *Prevotella* genus showed a negative correlation with G2 and G3. Firmicutes/Bacteroidetes showed positive correlation with G3 and G1.

**Wan et al., 2022 [26]**	1-month-old male KM mice	G1: Control group (n = 15) G2: Running group(n = 15)	4 weeks	Voluntary running (Free access to running wheel)	↔ ACE, Chao and Shannon index. ↑ Abundance of Genus: *Parabactoids, Anaerovorax,* Order: *Clostridiales* in G2; Abundance of Genus: *Anaerotruncus* and *Odoribacter* in G1. ↓ Genus: *Intestinimonas* on G2. * Beta diversity analysis showed differences between the communities of the groups.

**Yun et al., 2022 [29]**	4-wk-old female C57BL/6 mice	G1: Chow diet group (n = 5) G2: Chow diet plus exercise group (n = 5)	8 weeks	Treadmill running (Intensity: 15–20 m/min, 5% grade); 60 min/session; 1 session/day; 5 days/wk.	↔ Firmicutes and Bacteroidetes abundance; Beta diversity. ↑ Cyanobacteria in G2. ↓ *Paraprevotellaceae* family in G4. * Beta diversity analysis showed differences between the communities of the group.

ACE: Abundance Based Coverage Estimator. AET: Aerobic exercise training. FTR: Forced treadmill running. SED: Sedentary. VWR: Voluntary wheel running. $\dot{\text{V}}0_{2\max}$: Maximal oxygen consumption. wk: week.w

### Analysis of Positive and Negative Effects

To evaluate the impacts of physical exercise on intestinal integrity and gut microbiota composition, a proportional scale was used to measure the positive and negative effects reported in each study, quantified based on the proportion of beneficial or harmful changes reported. Both effects were calculated as proportions ranging from 0 to 1, with the sum of positive and negative effects for each study equaling 1.

Positive effects were defined as changes associated with benefits to intestinal health, including increased microbial diversity, higher abundance of beneficial bacteria, improvements in intestinal barrier integrity, and other markers indicative of functional or structural improvements. Negative effects, on the other hand, included changes associated with potential harms, such as an increase in bacteria linked to dysbiosis, a reduction in microbial diversity, or alterations in physiological parameters negatively impacting intestinal health.

The results were expressed as proportions of positive and negative effects for each study and presented in bar charts. Each bar reflects the proportional sum of beneficial and harmful changes reported in a single study, ensuring that the combined values of positive and negative effects always equal 1.

### Risk of bias

The SYRCLE Risk of Bias (RoB) methodology was used to assess the quality of the included studies [17]. This assessment was carried out independently by the two authors, considering the SYRCLE checklist, composed of 9 items that assess various types of bias (attrition, selection, detection, reporting, and performance bias, among others). The answers to each item are given by yes, no, and uncertain, which express, respectively, low risk (+), high risk (-) and unclear (?) risk of bias. Cochrane’s Review Manager software version 5.4 was used to produce the figure with each study’s final risk of bias.

## RESULTS

### Selection of studies

**FIG. 1:**
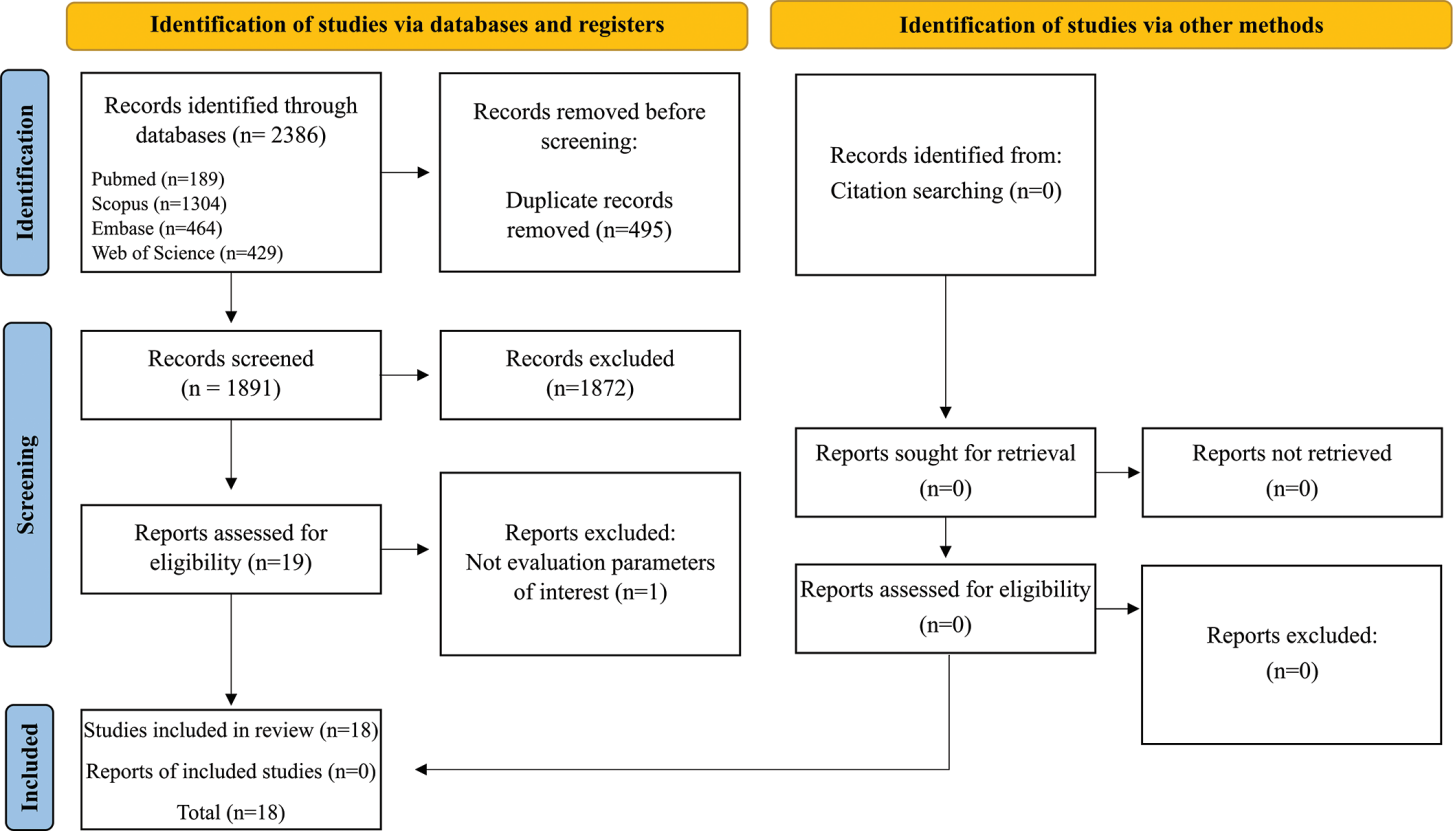
PRISMA diagram. Different phases of the selection of studies for conducting qualitative and quantitative analyses. Flow diagram of the systematic review literature search results. Based on Systematic review.

A total of 1,928 articles were identified. Then, 465 duplicates were removed, resulting in 1,463 articles, of which 1,444 were excluded after reading the titles and abstracts. After reading the articles in full, we excluded 1 article that did not evaluate the markers of interest. Thus, 18 articles were included in this review (Figure 1).

### Characteristics of the included studies

Out of the 18 articles included in our review, 13 (72.2%) used male animals (7 with rats and 6 with mice) [2, 12, 18–28], one (5.5%) used female mice [29], two (11.1%) used mice of both sexes [30, 31] and two (11.1%) did not describe the sex of the mice used [10, 32]. CB57/BL6 mice (n = 6) and Wistar rats (n = 4) were the two more commonly used species; the rest of the studies (n = 8) used Sprague Dawley rats, Fischer F344 rats, Kunming mice, ICR mice, C57/WT, KM mice, and C57BL/KsJ mice. The age of the evaluated animals ranged from 24 days to 12 months, and the duration of the studies from 6 days to 24 months (Table 2).

Regarding the type of physical exercise performed by the animals, in 16 out of the 18 selected articles, the effects of exercise were evaluated using treadmill running (n = 10) or running wheels (n = 6). One study evaluated the effect of resistance exercise (i.e., ladder climbing) [2], and another tested the effects of swimming [28]. Notably, in all studies, a control group was present that did not practice any physical exercise. In addition, in all studies, both animals that practiced physical activity and those that did not were given the same type of diet (Table 2).

Out of the 18 included articles, 16 (89%) used PCR of the 16S rRNA gene as the method for analyzing the gut microbiota. In contrast, 2 (11%) performed the analysis of the gut microbiota through specific culture media.

### Alpha diversity

**FIG. 2:**
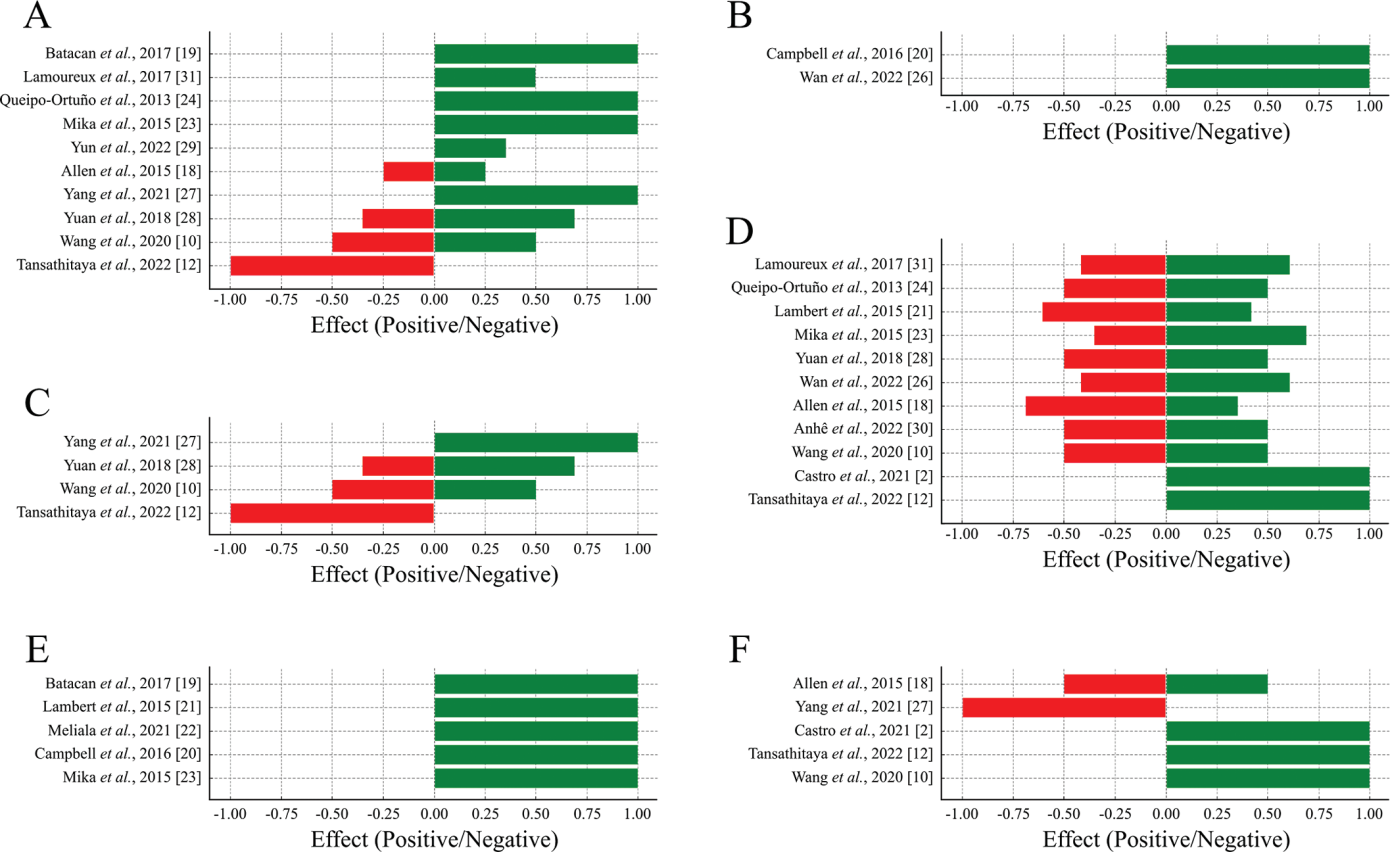
Proportion of positive and negative effects of physical exercise on alpha diversity. Positive effects (green bars) represent beneficial changes, and negative effects (red bars) represent potentially detrimental changes to gut health. (A) Phylum, (B) Order, (C) Family, (D) Genus, (E) Species and (F) Richness and diversity indices.

Out of the 18 articles selected, 16 evaluated the alpha diversity according to the abundance of phyla, orders, families, genera, and species, and also through richness and diversity indices. Overall, a predominance of positive effects was observed across all levels of alpha diversity analyzed, suggesting that physical exercise enhances microbial diversity and contributes to the stability of the intestinal ecosystem. Although negative effects were identified to a lesser extent, they were concentrated in specific parameters (Figure 2A–E). The richness and diversity indices further support this positive trend, highlighting the role of physical exercise in promoting intestinal health by improving microbial balance and diversity (Figure 2F).

### Phylum

The phylum level was evaluated in 11 articles, of which 4 observed an increase in Actinobacteria after voluntary exercise [23, 24, 31] and low-intensity exercise [19].

Five articles observed an increase in the abundance of Bacteroidetes after voluntary freewheeling exercises [23, 24, 31], swimming [28] or treadmill running [27]. It is noteworthy that one study reported this increase only in trained young animals [23]. In three studies, no differences were observed in the abundance of Bacteroidetes, either during voluntary or forced training [18] or when comparing forced exercise with sedentary exercise [29, 31].

Six articles evaluated the abundance of Firmicutes, and 3 of them found a decrease in the Firmicutes phylum in groups that underwent freewheeling training [23, 24] or forced training on a treadmill [31]. The decrease was observed in the young trained group in one study [23]. Two articles reported an increase in Firmicutes, specifically concerning freewheeling [31] and swimming [28]. Two studies found no significant change in the abundance of Firmicutes between groups [18, 29].

Two studies analyzed the Firmicutes/Bacteroidetes ratio. One of them reported a positive correlation between this ratio and heavy treadmill training compared to the sedentary group [12]. On the other hand, one study did not observe any changes in this ratio in trained animals [32].

In two articles, an increase in the abundance of Tenericutes was found in animals submitted to forced training [18] or that underwent low-intensity training [19].

Five studies reported the presence of the Proteobacteria phylum. In 3 of these studies, a decrease in abundance was observed in trained young animals [23], animals that swim [28], and animals that underwent high-intensity water training [10]. In one study found an increase in Proteobacteria in animals submitted to forced training [18]. In another study, did not observe any difference in this phylum between the animals that performed voluntary exercise and the sedentary group [31].

Three other phyla were observed in three studies. In one of them it was reported an increase in the phylum Euryarchaeota in trained young animals [23]. Another study observed an increase in TM7 in the group that performed high-intensity training [10]. The third study found an increase in Cyanobacteria in the trained group [29].

Furthermore, it is noteworthy that one study found no differences in the microbiota composition of trained and untrained animals [19].

### Order

Three studies evaluated the composition of the microbiota at the order level and observed increased *Clostridiales* in the trained group in freewheeling for 12 weeks [20] and voluntary exercise for 55 days [31] and 4 weeks [26]. Furthermore, when only the response to forced running on a treadmill was evaluated, a decrease in *Clostridiales* and *Lactobacillales* was observed.

### Family

Five studies assessed the microbiota composition at the family level. In one study, mice that exercised on a treadmill at 4.79 m/min for 60 min showed no difference in the *Enterobacteriaceae* family compared to the untrained group [21]. However, in another study, Wistar rats that ran on a treadmill at 8 m/min for 125 min showed an increase in *Erysipeloterichaceae, Bifidubacteriaceae,* and *Coreobacteriaceae* [19]. Another study found that animals undergoing voluntary exercise showed no change in *Bacteroidaceae* compared to the control group [31]. However, despite this, voluntary exercise increased the abundance of the S24-7 family and decreased the abundance of *Rikenellaceae*.

In one study, observed that animals submitted to two daily swimming sessions until exhaustion showed an increase in the abundance of the *Helicobacteriaceae* family and a decrease in *Bacteroidales_S24-7_group* and *Lachnospiraceae* [28]. Additionally, another study found that elderly mice undergoing aerobic exercise reduced *Lachnospiraceae* family compared to sedentary elderly mice; in this same group, a decrease in *Turicibacteriaceae* abundance was also reported [30].

### Genus

Of the selected studies, 3 evaluated the abundance of *Bacteroides,* and 2 reported an increase in the abundance of this genus following either forced exercise on a treadmill [31] or swimming [28]. In contrast, another study found that the non-exercised group had an increased abundance of the genus *Bacteroides* [24].

The genus *Prevotella* was evaluated by 2 studies, which presented similar data. In one study, the abundance of the *Prevotella genus* decreased with increasing exercise intensity, both in light and intense exercises, compared to the absence of physical exercise [12]. In another study, a higher abundance of *Prevotella* was observed in untrained animals [24]. Additionally, in a separate study, rats subjected to moderate to intense running exhibited a reduced *Bacteroides/Prevotella* ratio compared to sedentary rats [21].

One study observed an increase in the *Turicibacter* abundance when adult animals actively engaged in wheel activity [23], and another study observed a decrease in *Turicibacter* in animals that performed voluntary exercise compared to sedentary animals [18]. As for the genus *Odoribacter*, the selected studies reported contradictory results. In one study a decrease in this genus was observed in animals that practiced swimming for 4 weeks [28]. However, in another study, voluntary exercise led to its increase [26]. Furthermore, in the latter study, an increase in the abundance of *Dorea* and *Anaerotruncus* was also observed, which aligns with the results found in another study [18].

Moreover, among the selected studies, 4 assessed the impact of voluntary exercise on the microbiota, revealing that physical exercise increased the abundance of the following genera: *Clostridium, B. Coccoides-E rectale, Blautia spp, Anaerostipes spp, Methanosphaera spp, Rikenellaceae g_AF12, Rikenellaceae g_, Parabactoids* and *Anaerovorax* [23, 24, 26] Additionally, it was found that physical exercise reduced the abundance of *Enterococcus, Rikenellaceae g_, Rikenellaceae g_AF12, Desulfovibrio spp, Allobaculum, Anaeroplasma, Akkermansia, Eubacterium,* and *Intestinimonas* [23, 24, 26, 30].

When the animals underwent forced treadmill training, researchers observed an increase in the abundance of *Dehalobacterium* and a decrease in *Methanobrevibacter, Parabacteroides*, and *Candidatus arthromitus* [10, 21, 31].

Only one study evaluated the effect of resistance exercise on the gut microbiota, in which an increase in *Coprococcus_1,* and a decrease in *Pseudomonas, Serratia,* and *Comamonas* was found in animals manifested by resistance exercise [2].

### Species

Among all the articles analyzed by this review, 5 studies assessed the microbiota at the species level. Among these, 3 evaluated the effect of forced exercise on a treadmill [19, 21, 22], and 2 evaluated the effect of voluntary exercise on a wheel [20, 23].

Among the studies that evaluated forced exercise, increases in the species *Bifidobacterium spp, Lactobacillus spp, Clostridium leptum,* and *Clostridium cluster* (C-I) [21], *Parasutterella excrementihominis* and *Lactobacillus johmsonii, Clostridium saccharolyticum, Clostridium gelpurificans* were observed [19]. Furthermore, a reduction in *Escherichia coli* was observed [22].

Regarding voluntary exercise, we found no difference in the species composition of the gut microbiota between the sedentary and trained adult groups [23]. However, an increase in the number of species was observed when comparing adult animals with young ones. Additionally, there was a decrease in species in trained animals compared to sedentary ones. In one study an increase in *Halobacullum spp* and *Faecalibacterium prausnitzii* was observed in trained animals [20].

### Richness and diversity indices

In 7 studies, the Shannon and Chao indices were utilized to assess intestinal microbial richness and diversity in groups. The analysis of diversity using the Shannon index showed contradictory results among the selected studies.

In one study, both forced exercise on a treadmill and voluntary exercise did not alter the microbial diversity of mouse feces [18]. However, when this diversity was evaluated in the cecal content, the animals that underwent voluntary training increased this index. In contrast to this result, another found no difference in the Shannon index in mice submitted to voluntary training or not [26]. In another study, where mice were exposed to high-intensity intermittent exercise, an increase in diversity was also observed in the trained group.

Similar to the Shannon index, the Chao index also presents contradictory data among studies. In animals that exercised voluntarily, there was a reduction in the Chao 1 index [18]. A similar result was found one study where the sedentary group had a higher Chao index compared to the trained group [27]. In contrast, in two studies, which focused on resistance exercise and high-intensity treadmill running, there was an increase in the Chao index in trained animals [2, 12]. In a more recent study evaluating the effect of voluntary exercise on the gut microbiota, no differences were found in diversity using the Chao index [26]. Furthermore, in terms of species richness analysis, one study found no difference in species richness in a sample of intestinal mucosa between trained and untrained groups [31].

### Beta diversity

**FIG. 3:**
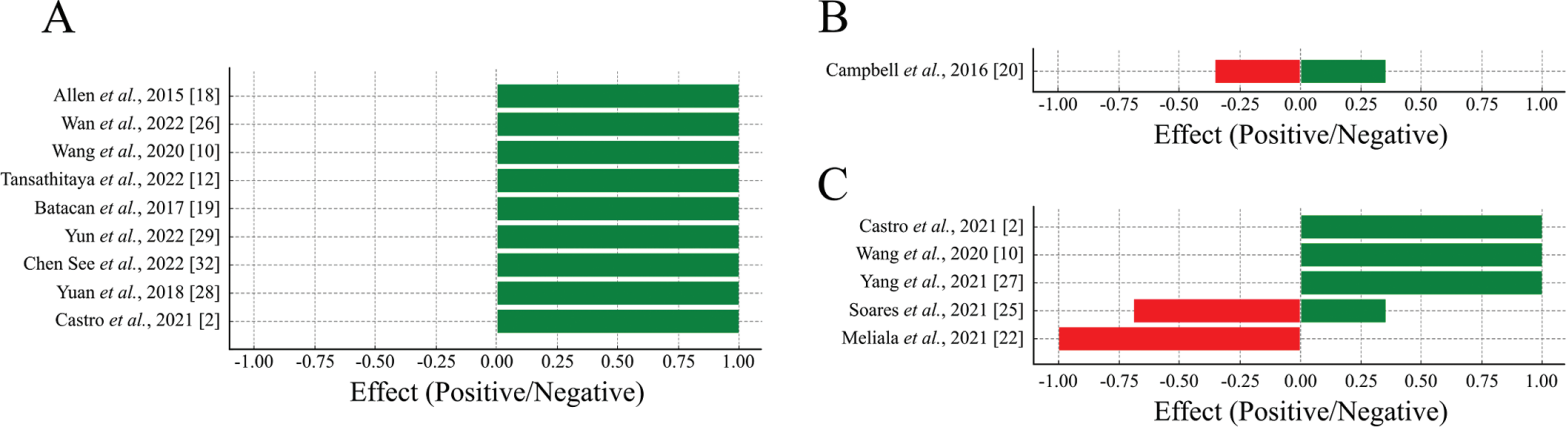
Proportion of positive and negative effects of physical exercise on beta diversity (A), mucosal integrity (B) and microbiota functionality (C). Positive effects (green bars) represent beneficial changes, and negative effects (red bars) represent potentially detrimental changes to gut health.

Among the included studies, 13 evaluated beta diversity. A predominance of positive effects was observed for beta diversity, indicating that physical exercise promotes greater differentiation and balance in the composition of the intestinal microbiota among the groups analyzed (Figure 3A).

Out of these, 5 studies examined the effect of voluntary exercise on intestinal microbial beta diversity. In 3 out of 5 studies, voluntary exercise did not impact microbial diversity between trained and untrained rats [23, 30, 31]. In two studies, the practice of voluntary physical exercise in a circle altered beta diversity [18, 26].

Eight studies analyzed the effects of forced treadmill exercise on microbial diversity. In 6 of these studies, running on a treadmill, whether light or heavy, continuous or intermittent, changed the gut microbiota of trained rats, resulting in the formation of distinct communities when compared to sedentary control [10, 12, 18, 19, 29, 32]. In one study found that rats that voluntarily exercised did not exhibit changes in beta diversity compared to the sedentary group [31]. Similarly, another study observed no changes in microbial diversity between the rats that underwent the training program and the control group [27].

Additionally, was observed a dissimilarity between bacterial communities in animals that underwent swimming compared to sedentary animals [28]. One study showed a difference in the beta diversity between animals subjected to vertical climbing for 12 weeks and sedentary animals [2].

### Mucosal integrity

The integrity of the mucosa is directly linked to the gut microbiota, which can modulate the expression of proteins associated with intestinal permeability and alter this organ’s morphology. An equilibrium between positive and negative effects was observed for mucosal integrity, suggesting that physical exercise has a nuanced impact on intestinal barrier function, reflecting a complex relationship between exercise and mucosal health (Figure 3B).

In one study, an increase in duodenal villi thickness and a decrease in E-cadherin were observed in trained animals. However, the expression of occludins and COX 2 showed no significant difference between the trained and sedentary groups [20]. In two other studies that examined intestinal morphology after physical exercise (swimming or running on a treadmill), no discernible differences were observed in the structures of the small intestine and colon in these animals compared to the untrained group [25, 28].

### Functionality of the microbiota

A predominance of positive effects on microbiota functionality was observed, indicating that physical exercise enhances the functionality of the intestinal microbiota. Negative effects were minimal, suggesting that exercise generally has a beneficial impact on microbiota functionality (Figure 3C).

Two studies observed a decrease in lipid and amino acid metabolism, cellular processes, and signaling in resistance training compared to the sedentary control group [2, 10]. Additionally, a reduction in genes associated with diseases, such as obesity, was observed in the high-intensity interval training group compared to the control group [10].

Physical exercise increased the functions of the microbiota related to carbohydrate metabolism and decreased the metabolism of lipids and amino acids [2, 10]. In a study, a positive correlation was observed between the practice of moderate-intensity physical exercises and the enhancement of metabolic activity in the gut microbiota [27].

Trained rats exhibited increased production of propionic acid in both feces and the intestine while showing reduced production of acetic acid in the feces and succinic acid in the intestine compared to the sedentary group [25]. Similar findings were observed in rats submitted to high-intensity exercises, where the production of SCFA decreased compared to the control group [22]. However, in the same study, no significant differences were observed in SCFA production between the moderately trained and sedentary groups when evaluating the effect of moderate physical activity. Additionally, there was no difference in lactic acid bacteria among all the groups evaluated in the study.

### Risk of bias

**FIG. 4:**
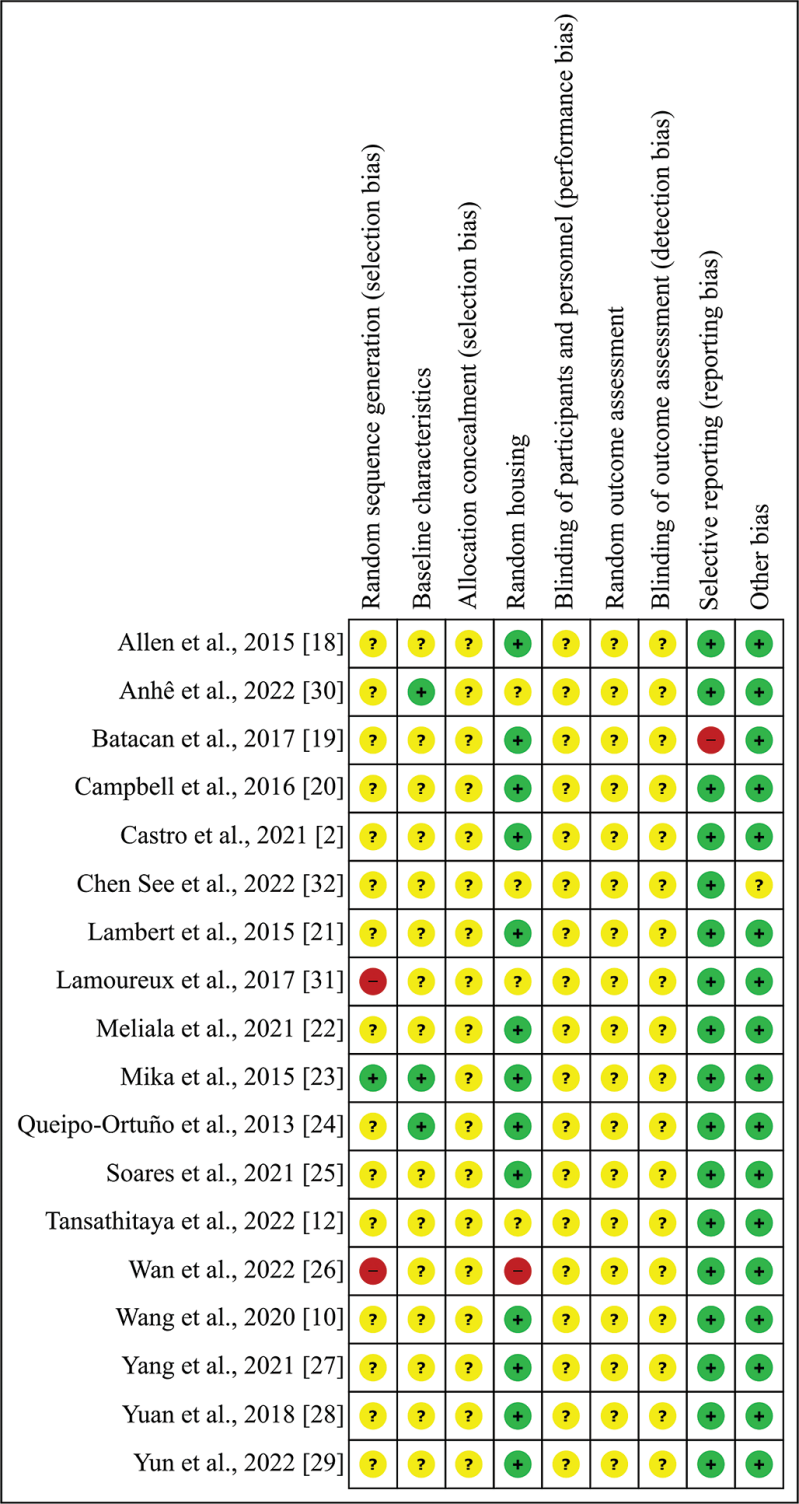
Risk of bias assessment: Summary of authors’ judgments on the risk of bias item for each included study. Articles were scored with “yes” indicating a low risk of bias, “no” indicating a high risk of bias, or “unclear” indicating that the item was not reported, according to the Center for Systematic Review for Experimentation with Laboratory Animals parameters. (SYRCLE). Green (+): Low risk of bias; Yellow (?): Possible risk of bias; Red (-): High risk of bias.

In the studies included in this systematic review (n = 18), the random component in the sequence generation process was described in only one article (5.56%), and the baseline characteristics were mentioned in three articles (16.67%). Allocation concealment, caregiver and investigator blindness, random selection for outcome assessment, and rater blinding were not described in all articles (100%). Random allocation of animals was reported in 13 studies (72.22%); in one study, not all pre-specified primary outcomes were reported (5.56%), and finally, one study did not make it clear that it was free from other problems that could result in a high risk of bias (5.56%) (Figure 4).

## DISCUSSION

This systematic review aimed to differentiate itself by addressing in detail the influence of physical exercise on the gut microbiota. Unlike other studies, we sought to highlight the variations between the types of exercise, whether voluntary or forced, and how these methodological choices impact the results due to the potential influence of stress and exercise intensity on the animals studied.

The studies revised here suggest that regular physical exercise has a significant impact on the gut microbiota [18, 23, 30, 31]. Moreover, such impact is highly variable according to the type and intensity of the exercise performed and the age of individuals [23, 33].

Several studies have shown that most human diseases, such as obesity, diabetes, and psychiatric diseases, affect the diversity of the gut microbiota [34–37]. Recent findings further highlight the relationship between physical activity and gut microbiota. For instance, higher levels of physical activity are associated with greater fecal microbiota diversity and improved composition, emphasizing the beneficial effects of physical activity on gut health [38]. Similarly, significant differences in microbiota profiles across sedentary individuals and soccer players, showcasing the modulating effect of exercise intensity on microbial composition [39]. Additionally, regular physical activity could shift gut microbiota structure in aged individuals with metabolic syndrome, suggesting that these changes may mitigate the adverse effects of aging and obesity [40].

**FIG. 5:**
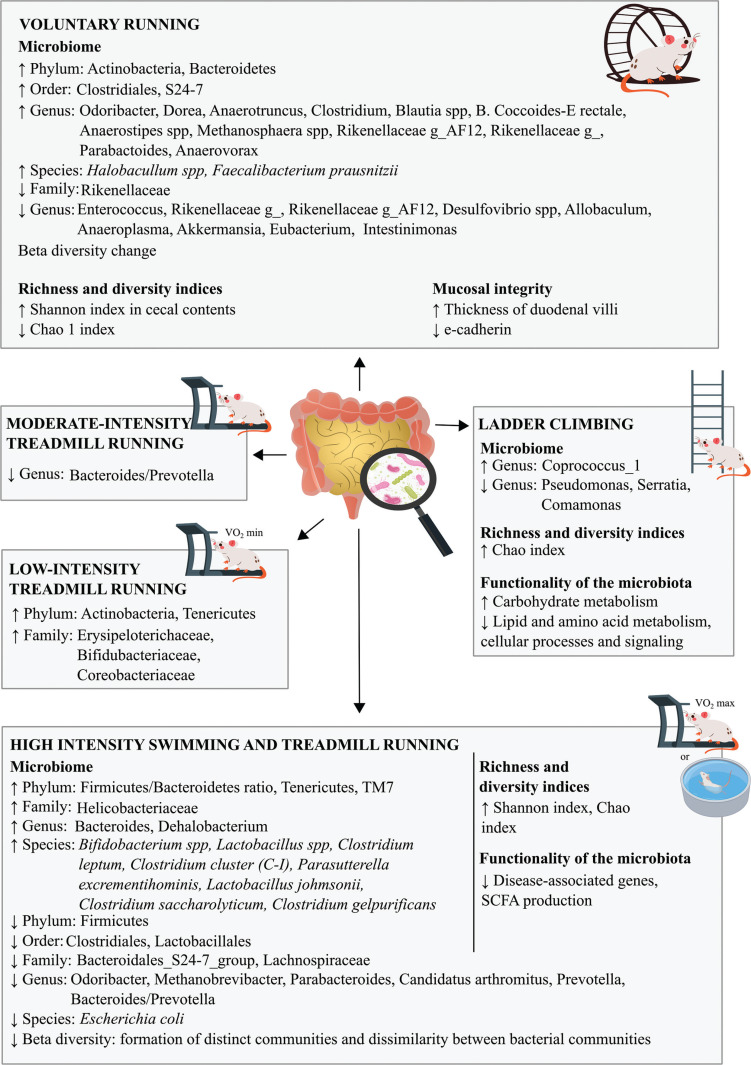
Impacts of physical exercise on the microbiota in an animal model.

Thus, the practice of different types of exercise, either forced or voluntary, as a strategy for modulating the composition of the gut microbiota is of paramount importance for maintaining good microbiological diversity, as well as its stability, which is associated with a healthier status [23, 28, 41] (Figure 5).

Among the most interesting alterations observed in this review was the increase in the abundance of the Phylum Firmicutes and the Firmicutes/Bacteroidetes ratio after training. This data is controversial since the increase in Firmicutes as well as in the Firmicutes/Bacteroidetes ratio has been correlated with an obese and unhealthy phenotype [42], due to its greater efficiency in extracting calories from food, promoting a more efficient absorption with consequent weight gain [43, 44].

Despite that, these results must be evaluated with caution since the microbiota analysis at the phylum level presents a very broad character; therefore, it becomes dangerous to characterize an entire phylum as bad. For example, *Clostridium leptum*, belonging to the Firmicutes phylum, is related to increased production of butyrate and SCFA, which has beneficial functions such as improvement of the intestinal epithelium, and protection against pathogens and intestinal diseases [24, 45, 46]. Thus, the elevation of butyrate-producing Firmicutes can be beneficial to the host [21, 47].

Two out of the 18 evaluated studies showed consensus on the role of regular physical exercise in increasing the abundance of the phylum Tenericutes [18, 19]. Despite that, the role of this phylum in the gut microbiota is still contradictory. Studies have already associated this phylum with the ingestion of high-fat diets and also ulcerative colitis [48, 49]; however, in another study, its abundance was reduced in colitis [50]. Therefore, deeper research must be carried out to understand its role in the homeostasis of the gastrointestinal tract.

When evaluating the taxonomic level of order, the *Clostridiales* call attention. Voluntary running increased the abundance of this order [20, 26, 31]. Bacteria belonging to this order produce SCFA [51], which can act in the prevention of obesity, colon cancer, and Crohn’s disease [52–54].

Regarding the family level, unexpected results were observed, in which the abundance of *Lachnospiraceae* was reduced in mice who underwent swimming or voluntary running protocols [28, 30]. This family of bacteria is known for its high production of butyrate, which has several health benefits [55]. It is hypothesized that a slight change in the inflammatory response can cause immunological and neuroendocrine changes that contribute to the alteration of the gut microbiota [56, 57]. This result, was corroborated by a study that associated *Lachnospiraceae* with a more inflammatory state [58].

When analyzing the results at the gender level, the increase in the abundance of *Bacteroides* seems to be related to the practice of enforced exercises (i.e., treadmill running and swimming) [28, 31]. In contrast, the practice of voluntary running does not have such an effect [24]. Bacteria belonging to this genus produce SCFA, mainly acetate and propionate, which help control energy metabolism [26, 59]. In a study, *Bacteroides acidifaciens* were related to the prevention of obesity, through the improvement of insulin sensitivity and energy metabolism [27]. This effect is likely due to acetate’s ability to promote insulin secretion [59].

The practice of physical exercise also altered beneficially the abundance of the genera *Prevotella* and *Turicibacter*, both of which can act in the modulation of the host’s immune response. *Prevotella* species can act on type 17 helper T cells and regulatory T cells, initiating an immune imbalance related to a high risk of rheumatoid arthritis [60, 61]. In a study carried out with *knockout* mice for Toll-like receptor 2, no *Turicibacter* population was detected, showing its relationship with the immune response [62].

Out of the selected studies, only 5 assessed the gut microbiota down to the species level. Among the species that had their abundance increased, *Lactobacillus spp.* stands out [21]. Additionally, it has been observed that probiotic supplements with *Lactobacillus* strains are associated with improved physical performance [63]. Despite that, the increase in the abundance of *Lactobacillus* was observed only with moderate to intense physical exercise, whereas at low intensity they may have the opposite effect [21, 31].

Alpha diversity analyses were performed using the Chao and Shannon indices. In general, the Chao index measures community richness, while the Shannon index assesses the richness and uniformity of a community. Among the selected studies, 4 had a test group that underwent voluntary running and observed reduced alpha-diversity in 2 of these studies [18, 27], while the other 2 studies showed no difference between exercised and control mice [26, 31]. However, when forced exercise was evaluated, it proved to be more efficient in changing diversity. For example, rats submitted to ladder climbing or treadmill running, showed an increase in alpha diversity compared to their non-exercised controls [2, 12].

Thus, as with alpha diversity, enforced exercise is more effective in modifying beta diversity. Studies have suggested that increased diversity is related to improved metabolic functions and general health status [64]. These effects may be related to the activation of intracellular AMP kinase caused by physical exercise. Activation of the AMP-activated protein kinase (AMPK) pathway leads to improved insulin sensitivity and metabolic regulation; it may exert an anti-inflammatory action that is reflected in the improvement of the microbiota [18, 65].

Regarding intestinal morphology, regular physical exercise, whether voluntary or forced, does not seem to lead to histologically visible changes [20, 25, 28]. However, in one study it was possible to find a reduction in E-cadherin in mice who had free access to running wheels [20]. This reduction can lead to increased intestinal permeability with possible health damage [66]. Nevertheless, these results should be analyzed with caution, since the mice did not receive an *ad libitum* diet, and prolonged fasting periods have already been shown to be related to increased intestinal permeability [67].

Given this, the discrepancies observed in the effects of physical exercise on the gut microbiota can be attributed to a series of factors, including the type of exercise (voluntary *vs.* forced), the intensity (light *vs.* high), and especially the impact of specific characteristics of the species used in the studies. The role of stress, particularly in forced exercise protocols, deserves special attention since different levels of stress can significantly alter the composition of the microbiota and intestinal integrity.

Although many studies have not reported these stress levels consistently, it is plausible that the response to stress varies not only between exercise protocols but also between species, such as rats and mice, which may explain some of the reported inconsistencies. Including objective measures of stress is essential in future investigations, as it will allow a more robust assessment of the effects of physical exercise in the context of the gut microbiota and its interaction with other biological factors.

Therefore, more experimental studies are needed to clarify the possible effects of different exercises on the gut microbiota. Future studies should focus on standardized and appropriate diets and animal models, and prioritize the use of metagenomic data, mainly through shotgun sequencing, for a deeper understanding of changes in the gut microbiota generated by regular physical exercise.

Thus, this review sought to explore not only the observed taxonomic alterations but also the potential underlying mechanisms, including modulation of intestinal permeability, immune regulation, and interactions among specific bacterial species. In this sense, our approach goes beyond simply reviewing existing data, providing a critical overview of the gaps in the literature, and highlighting priority areas for future investigations. This focus on methodological limitations and biological mechanisms reinforces the relevance of this work for advancing research in the field of gut health and physical exercise.

## Knowledge gaps, challenges, and future directions

The studies included in this systematic review point to the positive effects of regular physical exercise on intestinal health in animal models. However, there is a significant knowledge gap in this area, and additional research is needed to establish a solid scientific basis that can support recommending physical exercise as a means of improving gut health.

The interpretation of results in this review was challenging due to the scarcity of studies and the high variability in experimental designs and parameters evaluated. Variability included differences in exercise type (voluntary *vs*. forced), exercise intensity (mild *vs*. high), and species (rats *vs*. mice). These factors likely contributed to the discrepancies observed among studies. Future research should aim to standardize experimental designs and methodologies to reduce heterogeneity and facilitate comparisons. Additionally, the stress level induced by forced exercise protocols might confound results and should be carefully controlled and reported in future investigations.

Another critical challenge encountered is associated with the collection and analysis of microbiota data since the standardization of sampling, sequencing, and analysis methods is essential to ensure the consistency of results between studies. Heterogeneity in methods can make data comparison and synthesis difficult, hindering the construction of a solid overview.

Furthermore, it is important to understand the mechanisms by which physical exercise affects intestinal integrity and the microbiota, as although there is emerging evidence that regular physical exercise can have a positive impact on intestinal health, the mechanisms need to be elucidated in more detail, investigating how regular physical exercise affects the immune system, intestinal permeability, and interactions between different bacterial species.

Future research should address these gaps by adopting standardized methodologies, exploring mechanistic pathways, and controlling for confounding factors such as stress levels in forced exercise protocols. This will enable a more comprehensive understanding of the relationship between physical exercise and gut health, paving the way for evidence-based recommendations.

## CONCLUSIONS

The studies included in this review demonstrated that the benefits of regular physical exercise on the gut microbiota seem to be positively associated with training intensity. High-intensity swimming, ladder climbing, and treadmill running showed greater benefits in modulating the gut microbiota through increased richness and diversity without any change in intestinal morphology and integrity. However, this systematic review presents a high variability of data in the studies included, and more experimental studies are needed to clarify the possible effects of different physical exercise protocols on the gut microbiota.
